# Low-Cost, High-Fidelity 3D-Printed Temporomandibular Joint (TMJ) Dislocation Reduction Task Trainer

**DOI:** 10.7759/cureus.76938

**Published:** 2025-01-05

**Authors:** Richard Shin, Justin M Wang, Victor Huang

**Affiliations:** 1 Emergency Medicine, NewYork-Presbyterian Queens, Flushing, USA; 2 Emergency Medicine, US Acute Care Solutions, Lanham, USA; 3 Emergency Medicine, Northwell Health, Garden City, USA

**Keywords:** 3-d printing, emergency medicine, low-cost simulation, task trainer, tmj dislocation

## Abstract

Temporomandibular joint (TMJ) dislocation is an emergency that requires prompt reduction to relieve pain and restore function. However, practicing reduction techniques can be challenging due to the limited availability of real-world cases and high-fidelity task trainers. Hands-on TMJ dislocation reduction task trainers are not readily available, highlighting the need to improve traditional education methods to provide more effective and accessible hands-on learning opportunities. To address this gap, we developed a low-cost, high-fidelity three-dimensional (3D) printed TMJ dislocation reduction task trainer using open-source 3D computer-assisted design files. This innovative simulator provides a realistic, anatomically accurate model to teach and practice TMJ dislocation reduction techniques.

The task trainer was designed using 3D printing technology and inexpensive, readily available materials, significantly reducing production costs while maintaining high anatomical fidelity. The model replicates key anatomical features of the TMJ, including the mandibular condyles and articular surfaces, allowing for realistic hand placement, joint manipulation, and reduction procedures.

A stepwise approach was followed in the model's development, including iterative printing and validation through expert feedback from emergency medicine, oral and maxillofacial surgery, and simulation experts. The final product was evaluated in a pilot study, where resident physicians and medical students practiced reduction techniques on the trainer. Participants reported high satisfaction with the realism of the task trainer, particularly regarding tactile feedback and ease of manipulation.

With a 3D printer already available, cost analysis demonstrated that the materials for each task trainer cost less than $20. This cost-effective solution has the potential to democratize access to quality TMJ reduction training worldwide. In addition, the modular design of the task trainer allows for customization, offering opportunities for further advancements in medical simulation education.

Future areas of investigation include further validation studies to assess skill transfer to clinical settings and incorporation of this task trainer into emergency medicine and dental training programs. We believe that this low-cost, high-fidelity model will play a critical role in improving hands-on training for TMJ dislocation reduction, benefiting both learners and patients by enhancing clinical preparedness.

## Introduction

Spontaneous anterior temporomandibular joint (TMJ) dislocations are rare, with a reported annual incidence of 5.3 per 100,000 emergency department patients [1]. Although the incidence rate is low, TMJ dislocations are critical to address because they can affect airway secretion management, potentially result in airway obstruction, and cause significant pain for the patient [2]. Because of these reasons, healthcare providers need to understand the anatomical mechanics and subsequent dislocation reduction techniques of TMJ dislocations. Using a 3D printer, open-source design templates, and easily obtained materials within healthcare settings, we have developed a low-cost, reusable, and easily assembled model for simulated multidirectional TMJ dislocation reduction education.

At the time of project development, there are no simulation models of multidirectional TMJ dislocations described for use in medical education and simulation. With the use of this model, trainees are able to use various TMJ reduction techniques, including the intraoral, wrist pivot, and extraoral methods. The model will allow for the assessment of the appropriate traction force applied, tactile feedback for successful reduction, as well as direct visualization of the anatomical mechanics behind dislocation and reduction. Practitioners in emergency medicine, family medicine, dentistry, oral maxillofacial surgery, sports medicine, pediatrics, and primary care may benefit from the use of this simulated model. This report details the creation, assembly, and educational utility of a 3D-printed anterior TMJ dislocation model designed for use in simulation-based education (SBE) for healthcare providers. The model replicates the anatomical features of the TMJ and allows trainees to practice reduction techniques with realistic force application and resistance.

## Technical report

Materials and tools

**Figure 1:**
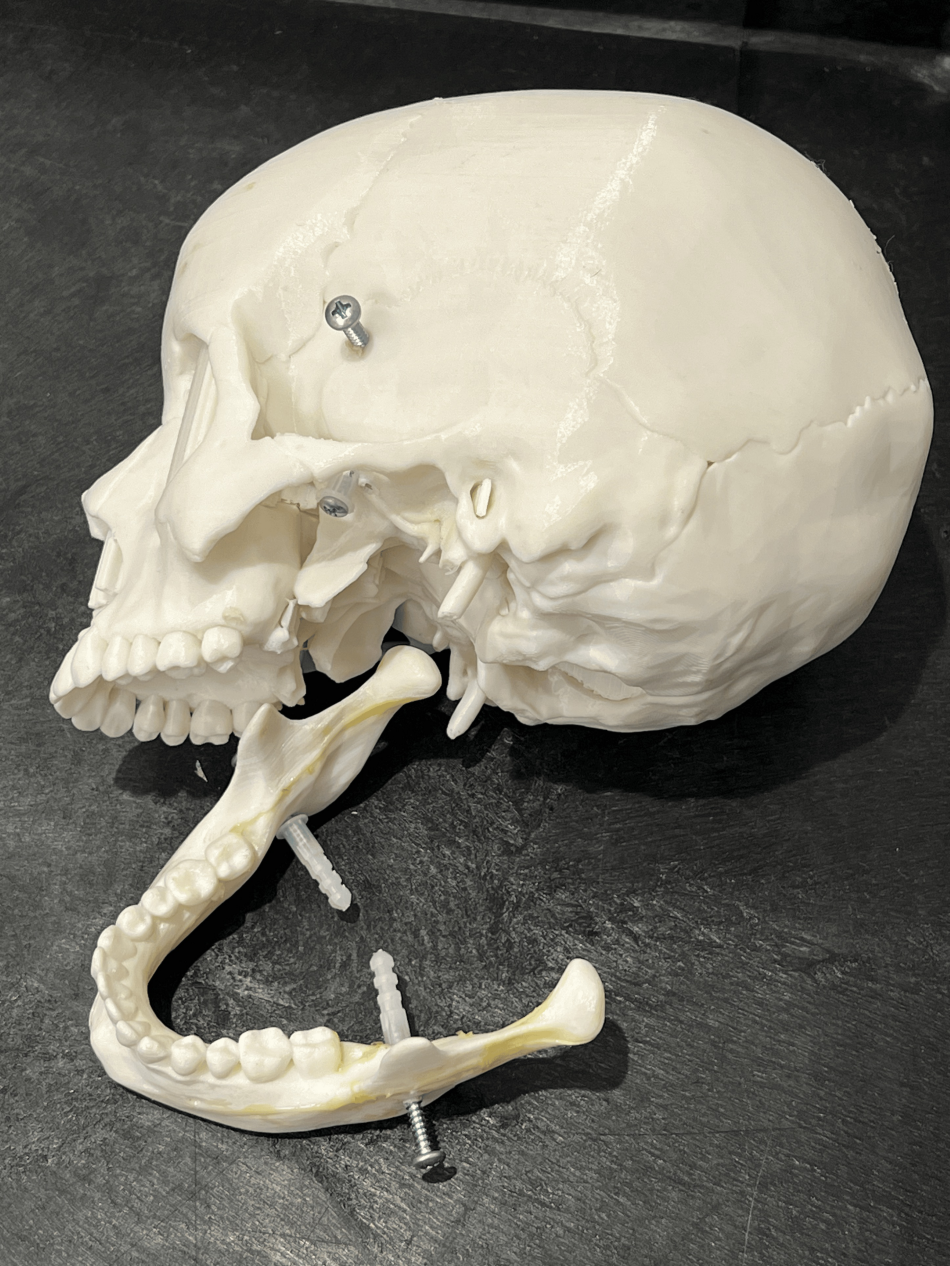
3D-printed model, jaw and skull with attachment posts for elastic bands

**Figure 2:**
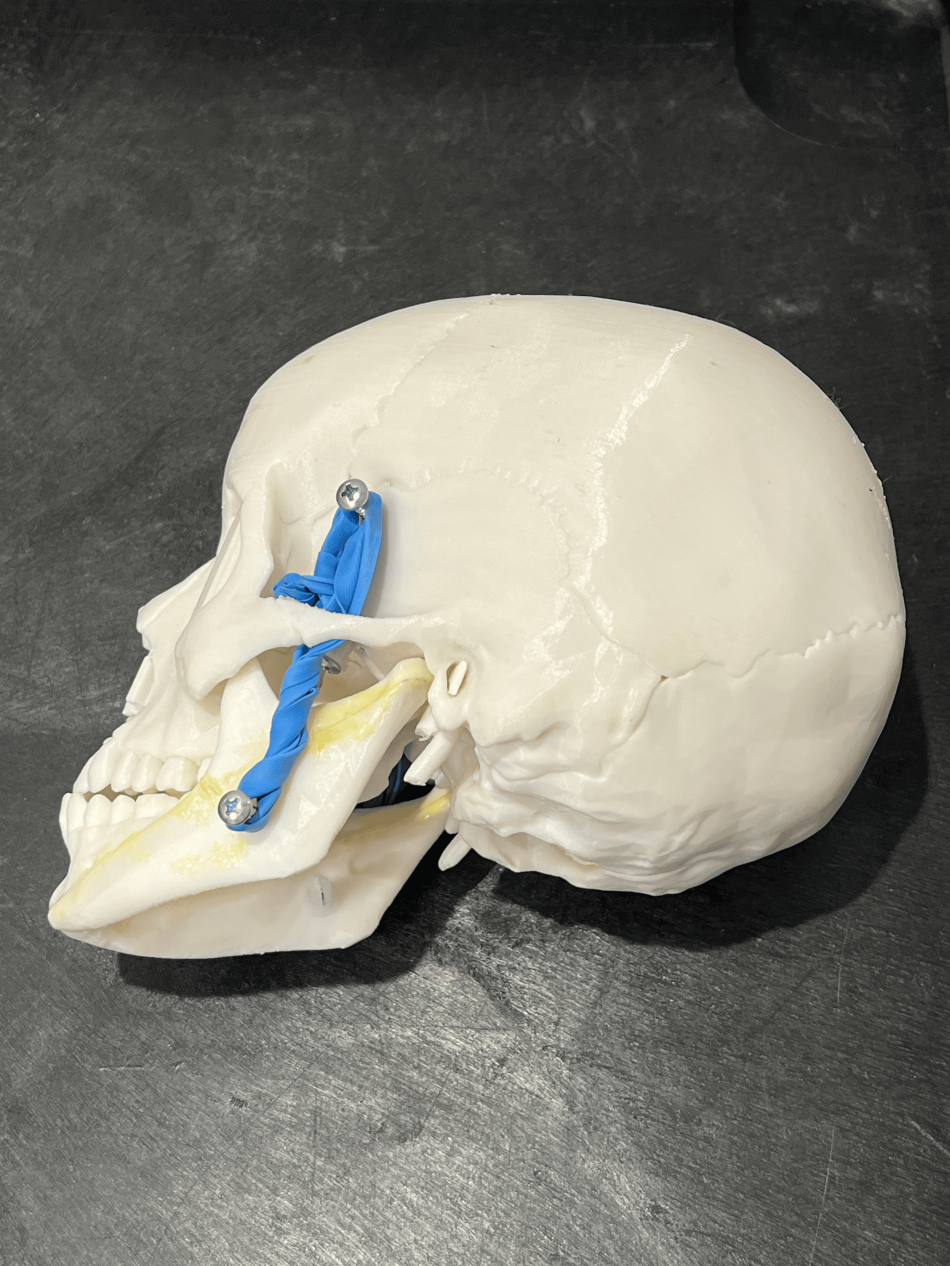
Fully assembled 3D-printed model

The primary tools required for constructing this model include a 3D-printed human-sized mandible model, superglue, self-tapping screws, and elastic bands. We 3D-printed a human skull and jaw utilizing the Creality Ender 3 Pro 3D® printer (Shenzhen Creality 3D Technology Co, Ltd., Shenzhen, China) and using a white polylactic acid (PLA) resin with templates from thingiverse.com [3], a 3D printing marketplace devoted to hosting and sharing of 3D models (Figure 1). Additional materials include self-tapping screws for mounting the elastic bands, which were screwed onto the mandible and lateral skull, and standard hospital elastic bands to simulate the dynamic muscle tension and tendinous insertions of the masseter and pterygoid muscles, which are involved in the reduction of TMJ dislocations (Figure 2). The model took approximately two days to complete the 3D printing process, though this duration may vary based on printer specifications, followed by about one hour of active assembly after printing.

**Figure 3:**
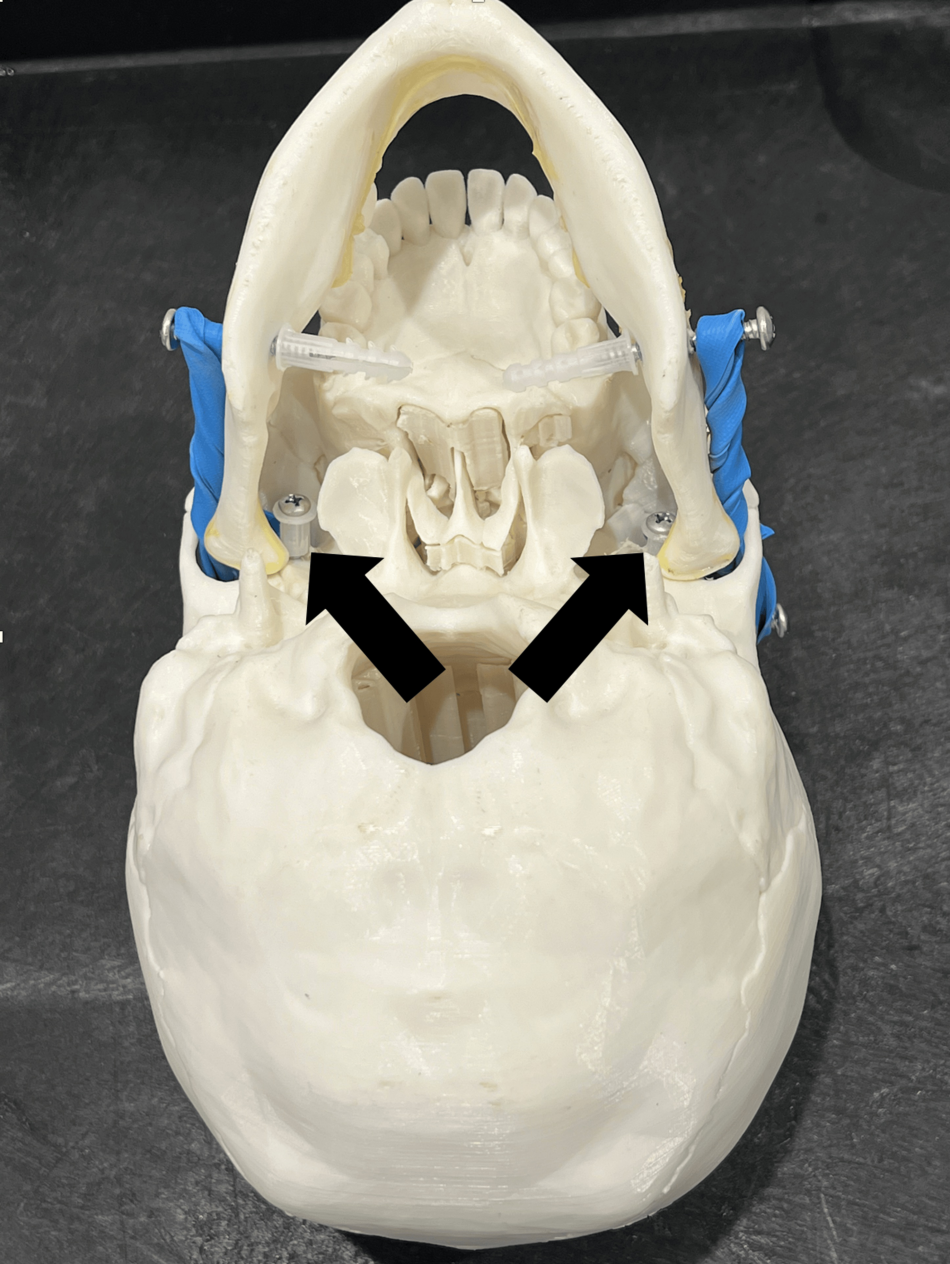
Additional screws added to prevent forward movement of the mandible

The assembly process begins once the 3D-printed components - specifically the skull and mandible - are completed. The mandible was printed in two parts and bonded with cyanoacrylate glue. Screws are inserted into the zygomatic arches bilaterally, serving as anchor points for the resistance mechanism. Additional screws are placed into the posterior and inferior aspects of the mandible to secure it to the skull, simulating the natural articulation and the tendinous insertion sites of the muscles surrounding the TMJ. To further replicate the biomechanics of anterior dislocation, screws are inserted anterior to each TMJ to help align the anatomic position of the mandible (Figure 3). 

**Figure 4:**
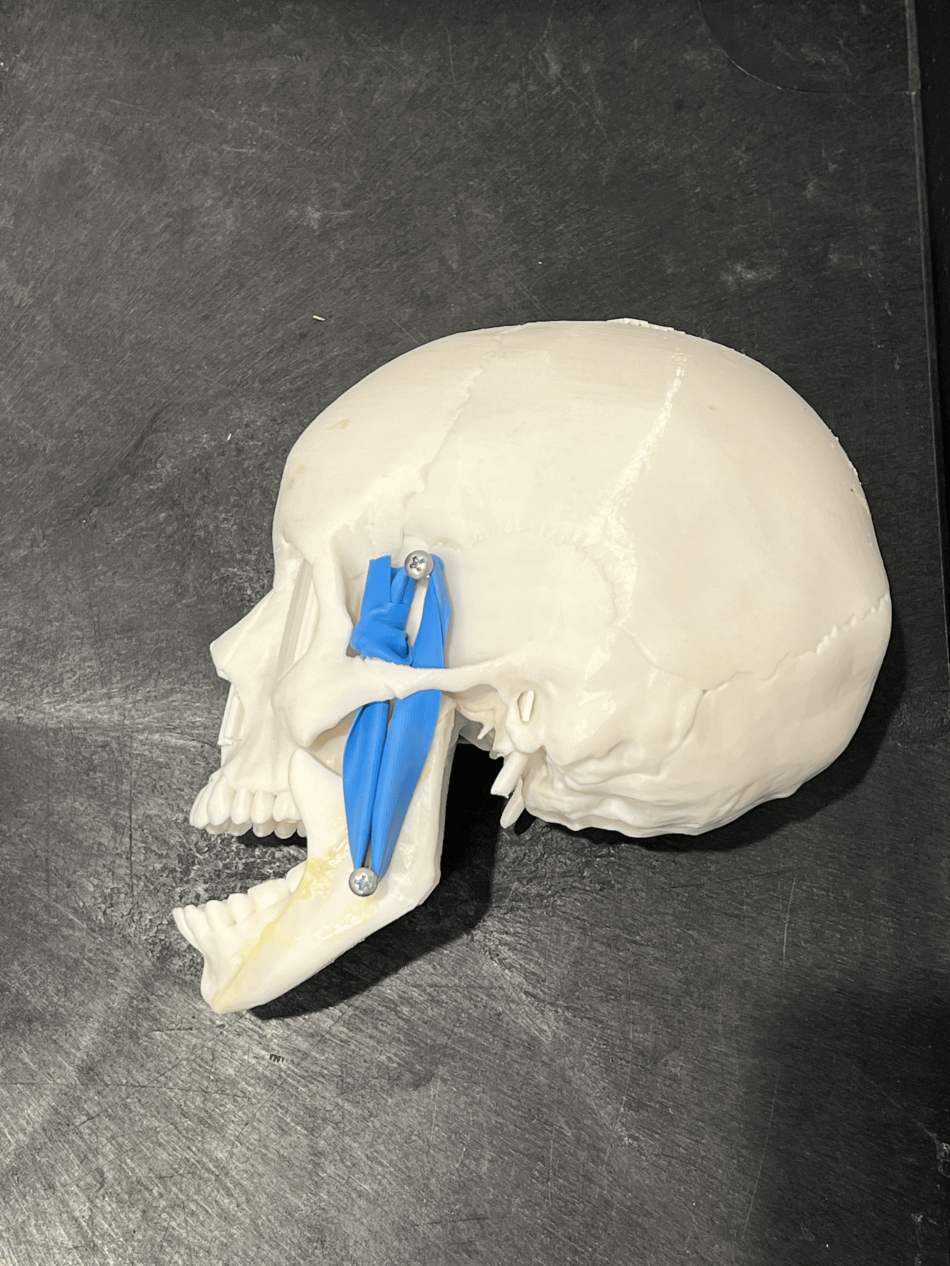
Anterior mandible dislocation on the 3D-printed model

To simulate the physiological resistance encountered during manual reduction, elastic bands are wrapped around the screws on each side, mimicking the muscle tension that must be overcome during the reduction maneuver. The tension can be adjusted, offering variable resistance to simulate different clinical scenarios or patient presentations, including multiple orientations of TMJ dislocations (Figure 4).

Educational utility and model functionality

This 3D-printed model provides an authentic platform for simulation-based medical education and deliberate practice in TMJ reduction techniques. The adjustable tension created by the elastic bands allows learners to experience the force required to reduce a dislocated mandible, facilitating the acquisition of psychomotor skills in a controlled environment.

In terms of learning outcomes, this model can be integrated into formative assessments, offering an opportunity for competency-based education in TMJ dislocation management. It provides immediate feedback to learners, promoting reflective practice, skill repetition, and gradual improvement. The model is also well-suited for interprofessional education, enabling collaboration between medical, dental, and emergency medicine teams to enhance team-based care during TMJ dislocation scenarios.

By incorporating this model into curricula for emergency medicine, oral and maxillofacial surgery, and family practice care providers, educators can create immersive simulation scenarios that mirror real-life clinical encounters, fostering critical thinking, decision-making, and procedural proficiency.

## Discussion

The 3D-printed TMJ dislocation reduction task trainer has the potential to offer an innovative and practical solution for procedural training, addressing a gap in hands-on medical simulation education. By leveraging affordable materials and 3D printing technology, this model provides learners with realistic, hands-on practice in a controlled, simulation-based environment. However, while the trainer is a significant advancement, there are limitations and areas for improvement that warrant further discussion.

One key limitation is the dependency on proper assembly and positioning of components to ensure the model’s functionality. The placement of screws and the alignment of the mandible are essential to replicate the joint’s natural articulation. Inaccuracies during the assembly process could potentially compromise the training experience, leading to a less realistic simulation of TMJ mechanics. As such, educators must ensure that assembly instructions are meticulously followed. In the future, there is potential to modify the open-source computer-assisted design file to include locations of anchor points for those interested in utilizing this task trainer. 

Another important consideration is the durability of the materials used. The elastic bands or tourniquets that simulate muscle tension are subject to wear and tear, particularly with repeated use in high-frequency training settings. Over time, the loss of elasticity may reduce the resistance that is critical for replicating the tactile experience of reducing a dislocated mandible. To address this, future iterations of the model could explore more durable alternatives, such as springs or synthetic elastomers, which may offer longer-lasting resistance and more accurate simulation of muscle tension dynamics. Furthermore, the durability of the PLA polymer used to create the structure of the 3D-printed model and its lifespan. 

Additionally, while the current model focuses on anterior TMJ dislocations, future versions could incorporate features that simulate other types of dislocations, such as posterior or superior dislocations. Expanding the model’s versatility to cover a broader range of dislocation scenarios would enhance its educational value and allow learners to practice more diverse reduction techniques. Moreover, integrating dynamic components, such as adjustable tension systems, could simulate varying levels of muscle rigidity, reflecting different patient presentations, such as those with spasms or chronic dislocation, further improving the realism of the model. Additional model changes that can enhance realism is to add a commercially supplied mask over the bony model to enhance realism for education. 

Cost-effectiveness is one of the primary advantages of this 3D-printed model, as it significantly reduces the financial barriers to accessing high-quality training tools. In general, most high-fidelity medical simulators are often priced in the range of several hundred to thousands of dollars, which makes them unaffordable for many educational institutions, especially those operating in low-resource settings. In contrast, the materials for this 3D-printed TMJ trainer cost less than $20 ($17 for commercially available 3D printing filament and one dollar for screws and adhesive), offering a highly affordable alternative without compromising educational value. This affordability can potentially improve access to quality medical education globally, particularly in resource-limited regions where such training tools are often unavailable.

Another area for future exploration is the model’s role in competency-based assessments. The realistic tactile feedback the model provides allows learners to practice reduction techniques repeatedly, promoting skill acquisition through deliberate practice. However, future research should focus on validating the model’s effectiveness in skill transfer to clinical settings. Longitudinal studies could assess whether repeated practice on this model translates to improved clinical performance, reduced time to reduction, and increased success rates in actual patient encounters. Such studies would be critical in establishing the model’s long-term impact on clinical preparedness and patient outcomes.

Interprofessional education is another area where the model could have significant applications. TMJ dislocation is a condition that may be encountered by a variety of healthcare providers, including emergency medicine physicians, dentists, oral and maxillofacial surgeons, and primary care providers. By incorporating this model into interprofessional simulation scenarios, teams of learners from different specialties could collaborate on TMJ reduction cases, improving communication, teamwork, and coordination during real-life clinical encounters. This interdisciplinary approach could enhance patient care by fostering a more teamwork-based approach to dislocation management across different healthcare fields.

## Conclusions

The 3D-printed model for anterior TMJ dislocation provides a versatile and accessible tool for medical educators to teach and assess procedural skills in TMJ reduction. Its low-cost, modular design and ease of assembly make it an ideal tool for SBE and skills labs across multiple disciplines. The model facilitates mastery of TMJ reduction techniques by simulating the tactile resistance encountered during dislocation which leads to enhancing learner confidence and competence.

Through simulation-based learning, deliberate practice, and competency-based assessments, educating using this model can significantly contribute to improving clinical outcomes in patients presenting with TMJ dislocations. By offering an accessible and reusable platform for procedural training, this model represents a step forward in the integration of 3D printing technology into medical education.

## References

[1] Lowery LE, Beeson MS, Lum KK (2004). The wrist pivot method, a novel technique for temporomandibular joint reduction. J Emerg Med.

[2] Chhabra S, Chhabra N, Gupta P Jr (2015). Recurrent mandibular dislocation in geriatric patients: treatment and prevention by a simple and non-invasive technique. J Maxillofac Oral Surg.

[3] Mel Mel, S. S. (2024). Human skull, anatomically correct and printer friendly updated with jaw [3D model]. https://www.thingiverse.com/thing:819046.

